# Connection between Systemic Inflammation and Neuroinflammation Underlies Neuroprotective Mechanism of Several Phytochemicals in Neurodegenerative Diseases

**DOI:** 10.1155/2018/1972714

**Published:** 2018-10-08

**Authors:** Jintang Wang, Yuetao Song, Zheng Chen, Sean X. Leng

**Affiliations:** ^1^Institute for Geriatrics and Rehabilitation, Beijing Geriatric Hospital, Beijing 100095, China; ^2^Division of Geriatric Medicine and Gerontology, Department of Medicine, Johns Hopkins University School of Medicine, Baltimore, MD 21224, USA

## Abstract

Oxidative damage, mitochondrial dysfunction, and neuroinflammation are strongly implicated in the pathogenesis of neurodegenerative diseases including Alzheimer's disease (AD) and Parkinson's disease (PD), and a substantial portion of elderly population at risk of these diseases requires nutritional intervention to benefit health due to lack of clinically relevant drugs. To this end, anti-inflammatory mechanisms of several phytochemicals such as curcumin, resveratrol, propolis, polyunsaturated fatty acids (PUFAs), and ginsenosides have been extensively studied. However, correlation of the phytochemicals with neuroinflammation or brain nutrition is not fully considered, especially in their therapeutic mechanism for neuronal damage or dysfunction. In this article, we review the advance in antioxidative and anti-inflammatory effects of phytochemicals and discuss the potential communication with brain microenvironment by improved gastrointestinal function, enhanced systemic immunity, and neuroprotective outcomes. These data show that phytochemicals may modulate and suppress neuroinflammation of the brain by several approaches: (1) reducing systemic inflammation and infiltration via the blood-brain barrier (BBB), (2) direct permeation into the brain parenchyma leading to neuroprotection, (3) enhancing integrity of disrupted BBB, and (4) vagal reflex-mediated nutrition and protection by gastrointestinal function signaling to the brain. Therefore, many phytochemicals have multiple potential neuroprotective approaches contributing to therapeutic benefit for pathogenesis of neurodegenerative diseases, and development of strategies for preventing these diseases represents a considerable public health concern and socioeconomic burden.

## 1. Introduction

With rapid population aging, advanced age is a major risk factor leading to an increased prevalence of neurodegenerative diseases including Parkinson's disease (PD), Alzheimer's disease (AD), amyotrophic lateral sclerosis (ALS), Huntington's disease (HD), and multiple sclerosis (MS). The common characteristic of these diseases is progressive neuronal loss and impaired neuronal function, and one of their critical backstage manipulators is extracellular neurotoxic microenvironment linked to oxidative stress, chronic inflammation, and mitochondrial dysfunctions [1]. These three situations not only possess their own signaling mechanism in neuronal loss, detrimental or beneficial, but also interact or crosstalk to promote neurodegenerative damage in a manner of vicious circle. Therefore, a better understanding of this pathological mechanism will help to develop therapeutic strategies for preventing or delaying disease processes. Currently, application of natural products in the prevention of these diseases is comparatively a new area, and supplementation of dietary phytochemicals is proven to be a promising nutritional intervention approach due to their neuroprotective properties such as antioxidation and anti-inflammation. Research shows that a wide variety of dietary phytochemicals, such as myricitrin, mulberry, and green tea, are endowed with antioxidative and anti-inflammatory features, and individual dietary habit determines availability of phytochemical types for health and therapeutic purposes, especially for the age-related diseases. The phytochemicals can consequentially increase overall physical quality and reduce neurodegenerative pathologies through at least three therapeutic attributions: gastrointestinal function improvement, immunity enhancement, and neuroprotective outcomes [2]. These attributions act independently or crosstalk to influence neural cell activities through immune network or neural network of peripheral and central nervous system (CNS), including vagal reflex pathway responsible for gastrointestinal signaling to the brain [3], inflammatory infiltration pathway through the blood-brain barrier (BBB) and gateway reflex [4], and direct neuroprotective regulations on glial cells and neurons within the brain [5–7].

Traditionally, parenchymal cells (i.e., neurons, astrocytes, microglia, and oligodendrocytes) of the CNS are separated from the rest of body by BBB to form an immune-privileged organ, and peripheral immune cells and nutrients such as resveratrol and curcumin are restricted into the brain. However, in recent years, substantial evidence shows that the brain itself is fully immune competent due to the participation of microglia and astrocytes in immune response [2]. Especially under pathological stimulation, peripheral immune cells such as monocytes and T and B lymphocytes can readily infiltrate into the brain through disrupted BBB or gateway reflex [8] and activate innate and adaptive immunity [2]. In addition, inflammatory reflex (i.e., vagal reflex or gut-brain axis) is another connection approach, by which systemic inflammation occurring in gastrointestinal tract or peripheral immune system can be sensed to form peripheral inflammatory signals that transmit into the brain to exacerbate chronic neurodegeneration [3, 7]. Likewise, dietary intake of these phytochemicals can form nutritional signals to reverse neuroinflammatory microenvironment by vagal reflex [4, 9]. As nutritional neuroscience is quickly growing, phytochemicals or nutraceuticals are proven to be important regulators of brain health and diseases, which can decrease production of proinflammatory cytokines and oxidative damage [10–12], and exert significant neuroprotective effect in neurodegenerative diseases [2]. Therefore, dietary nutritional intervention is beneficial for the elderly to remain both physically and cognitively healthy into older age or to prevent from neurodegenerative diseases, which represents a considerable public health concern and a potential solution to socioeconomic burden associated with rapid population aging.

In this article, based on the availability in daily life and reported evidence, we chose five phytochemicals, i.e., curcumin, resveratrol, propolis, polyunsaturated fatty acids (PUFAs), and ginsenosides, as representatives of various phytochemicals and discuss their neuroprotective mechanism and therapeutic implication for neurodegenerative diseases, including their anti-inflammatory and antioxidative effects on gastrointestinal dysfunction, peripheral immune system, and brain innate immunity, as well as potential communication of their nutritional signals between the brain and the periphery. The data about phytochemicals curcumin, resveratrol, propolis, PUFAs, and ginsenosides are collected from PubMed database and addressed in terms of antioxidative or anti-inflammatory mechanism and nutritional or protective effects on the gastrointestinal tract, systemic immunity, and neuroimmunity.

## 2. Oxidative and Inflammatory Mechanisms Underlying Neurodegenerative Diseases

**Figure 1:**
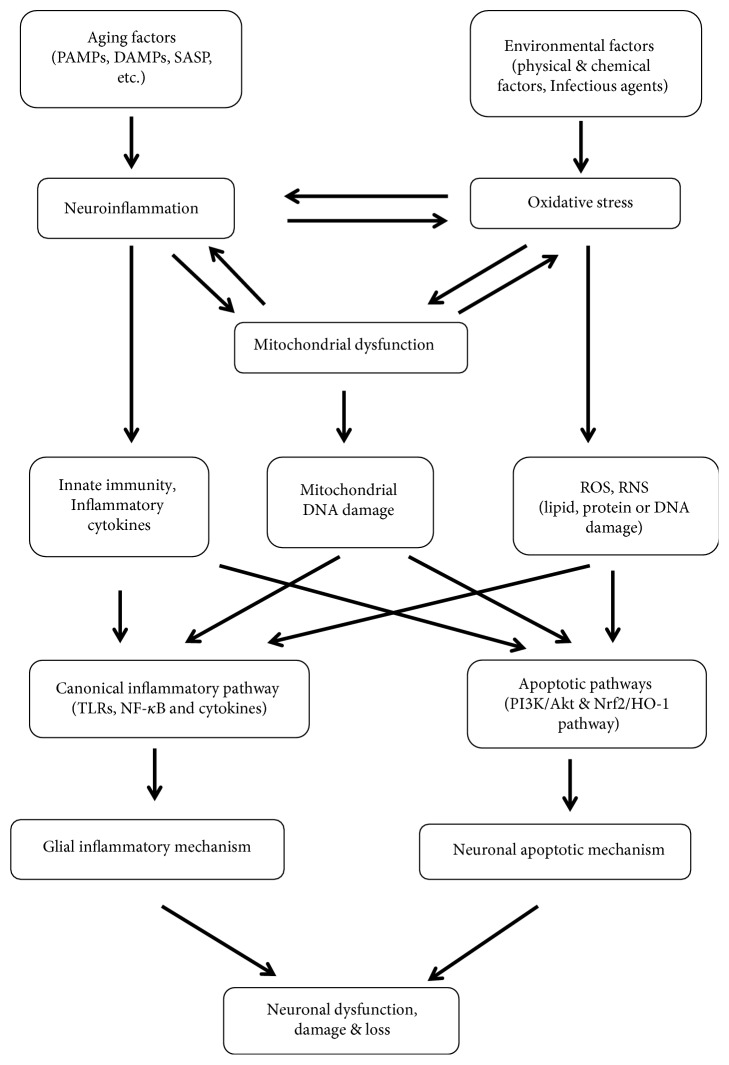
Correlation between oxidative stress, mitochondrial dysfunction, and neuroinflammation. Various aging factors and environmental factors stimulate glial cells to induce inflammatory response, oxidative stress, and mitochondrial dysfunction, which orchestrate to impact on neuronal apoptotic mechanism and glial inflammatory mechanism, leading to neuronal dysfunction or loss in neurodegenerative diseases.

Oxidative stress associated with mitochondrial dysfunction and neuroinflammation is a common characteristic of neurodegenerative diseases, mainly due to metabolic features of the CNS: high oxygen consumption even under basal conditions and high production of reactive oxygen species (ROS) and reactive nitrogen species (RNS) from specific neurochemical reactions, as well as increased metabolites such as pathogen-associated molecular patterns (PAMPs) and damage-associated molecular patterns (DAMPs) with aging [13, 14]. These three situations can interact with a causal relationship, as shown in Figure 1, to form a persistent stimulation to neuronal apoptosis and glial cell neurotoxicity, leading to neuronal dysfunction and damage or progressive loss [15].

Oxidative stress is defined as an imbalance between production of oxidants such as ROS and ability to detoxify reactive oxygen intermediates, causing cellular damage by free radicals. Normally, the brain function is highly sensitive to oxygen metabolic activity or production of ROS such as hydrogen peroxide (H_2_O_2_), hydroxyl free radical (∙OH), superoxide anion (O2^−∙^), and peroxynitrite (ONO_2_^−^), and approximately 98% of ROS is formed in mitochondria as by-products of cellular respiration [15]. Pathologically, various harmful factors, such as environmental factors (e.g., chemicals, UV light, and infectious organisms) and endogenous factors (e.g., dysfunctional mitochondria, abnormal enzyme activity, and aging factors), are accumulated to result in an imbalance between prooxidative and antioxidative reactions and subsequent oxidative damage (e.g., genetic mutations or epigenetic changes) to biomolecules (e.g., lipids, proteins, and DNA). These molecule alterations are proven to be the initiators of apoptotic mechanism linked to neuronal degeneration, involving apoptotic signaling pathway such as p53, caspase 3, caspase 9, Bcl2/Bax, Nrf2, and hemeoxygenase-1 (HO-1) and PI3K/Akt signaling pathway [16]. On the other hand, these molecules are also the activators of microglia and astrocytes to promote innate immunity and release of various proinflammatory cytokines such as tumor necrosis factor- (TNF-) *α*, interleukin- (IL-) 1*β*, interferon- (IFN-) *γ*, and IL-6 and form a neurotoxic microenvironment [7].

Chronic inflammation, another aging contributor, plays a critical role in neurodegenerative pathogenesis from initiation and progression to outcome of diseases, as a consequence of persistent stimuli of chronic stress antigens such as PAMPs, DAMPs, and senescence-associated secretory phenotype (SASP) [7, 17]. In the inflammatory process, these chronic stress factors stimulate microglia (resident immune cells) and astrocytes to activate canonical inflammatory pathway such as toll-like receptors (TLRs), nuclear factor- (NF-) *κ*B, and inflammatory cytokines IL-6, TNF-*α*, IL-1*β*, and IL-10 [18]. On the other hand, activated microglia is also an abundant source of free radicals in the brain to release excessive harmful ROS and RNS, etc., which in turn stimulate glial activation and innate immunity. Therefore, oxidative stress and neuroinflammation are two concomitant processes in aging and neurodegenerative diseases.

Mitochondrial dysfunction is a major source of ROS due to high energy demand and high dependence of brain activities on efficient mitochondria. It is readily affected by various environmental factors to occur early as a primary event in the aging process (e.g., mitochondrial DNA damage) and then is potentiated by microglia-mediated oxidative stress and neuroinflammation to fuel the pathogenesis of neurodegenerative disorders [14, 19]. For example, persistent oxidative stress and hypoperfusion in the brain can stimulate expression of nitric oxide synthase (NOS) to further drive formation of ROS and RNS and collectively contribute to BBB dysfunction and damage to brain parenchymal cells, and contrarily suppression of microglial activation can produce neuronal cell survival [1]. Taken together, oxidative stress, neuroinflammation, and mitochondrial dysfunction are orchestrated to form a neurotoxic microenvironment responsible for neuronal damage, and the underlying neuronal apoptotic mechanism and glial inflammatory mechanism will provide potential therapeutic targets for nutrients and phytochemicals to reverse pathogenesis of neurodegenerative diseases.

## 3. Gastrointestinal Health by Phytochemicals and Its Connection with Brain Innate Immunity via Inflammatory Reflex

Gastrointestinal function acts on gut microbiota, mucus integrity, gut immunity, and intramural neural plexus, and these functional components are readily influenced or damaged by gut inflammation and oxidative stress linked to various adverse factors. Especially, the gut damage, as a peripheral inflammatory stimulation signal, can be sensed by the brain through vagal reflex and exacerbate brain inflammatory response in the onset and progression of neurodegenerative diseases [7, 20]. Likewise, the improved gut function by phytochemicals is also sensed by vagal reflex to ameliorate neuroinflammatory response occurring in the brain. Therefore, neuroinflammation or neuroprotection may initiate from the periphery, and peripheral conditions powerfully influence various brain pathologies through vagal reflex or disrupted BBB [21].

Inflammatory reflex or vagal reflex, a bidirectional neuroimmune communication pathway between the gut and brain, also called gut-brain axis, consists of an afferent arm that senses peripheral inflammation and an efferent arm that sends out the signals integrated in the brain to inhibit gut inflammation and innate immune response. In detail, afferent signals in the vagal nerve, while reaching the solitary tract nucleus and brainstem, activate central neurons that project to the hypothalamus and other CNS nuclei responsible for inflammatory response control [22]. Namely, the brain perceives peripheral inflammation such as gut condition or gut microbiota dysbiosis through vagal afferent. For example, increased cytokine levels in the periphery and exogenous administration of proinflammatory cytokines such as IL-1*β*, IL-6, and TNFs can elicit sickness behavior of the brain, indicating that vagal afferent inflammatory signals mediate inflammatory response and relevant receptor activation within the brain [7]. Then, the integrated signals in the brain send a response to suppress immune system and regulate gastrointestinal function through vagal efferent [23, 24]. In other words, gut nutritional or microbial stimuli such as dietary intake of phytochemicals can activate the vagal nerve and send nutritional signals or nature of gut function to the brain to change neurochemistry of the brain and its behavior [25, 26]. Therefore, understanding reversal of gut inflammation and dysfunction by dietary phytochemicals and concurrent transmission alteration of these signals in vagal neural reflex may have an important implication for developing anti-inflammatory intervention and microbe- or nutrition-based therapeutic strategies for neurodegenerative diseases.

### 3.1. Curcumin

Curcumin is an oil-soluble polyphenolic phytochemical from *Curcuma longa* or turmeric and has an inhibitory effect on gut inflammation and gut permeability as evidenced in several preclinical and clinical studies. For example, curcumin protects gut function and metabolism by reducing chaperone BiP expression to modulate endoplasmic reticulum (ER) stress and inflammatory response in human intestinal epithelial cell lines, T84 and Caco-2 [27], or by increasing signals of neurotransmitters such as brain-derived neurotrophic factor (BDNF), serotonin, and cAMP response element binding (CREB) in the hippocampus and peripheral intestinal system [28]. In animal models, curcumin inhibits visceral nociception via antagonizing transient receptor potential vanilloid-1 (TRPV1) receptor, indicating a treatment implication for functional gastrointestinal disease [29]. In mice with TNF-*α*-induced ulcerative colitis, curcumin suppresses neutrophil priming and inducible NOS to counteract oxidative bowel damages from imbalanced gut immune response [30]. Meanwhile in the intestine, curcumin modulates dendritic cells to express aldehyde dehydrogenase 1a and IL-10 and induces differentiation of naive CD4+ T cells into regulatory T cells (Treg) to inhibit antigen-specific T-cell activation *in vitro* and reduce colitis *in vivo* [31].

### 3.2. Resveratrol

Resveratrol, a nonflavonoid plant polyphenol mainly found in red grapes and wine, possesses an anti-inflammatory effect to benefit gut health as evidenced in various inflammation models. In H_2_O_2_-induced Caco-2 cells, resveratrol increases epithelial expression of occludin and zonula occluden to protect gut barrier function and reduces intracellular ROS accumulation, along with increased expression of superoxide dismutase (SOD) and HO-1, to prevent oxidative damage [32]. In mice with ileitis, resveratrol downregulates T helper immune responses and increases Treg numbers and intestinal epithelial cell proliferation to maintain gut barrier function and prevent bacterial translocation [33]. In rats with colitis, resveratrol increases lactobacilli and bifidobacteria and inhibits enterobacteria to improve colonic mucosa architecture and reduce levels of colonic mucosa prostaglandin E2 (PGE-2), cycloxygenase-2 (Cox-2), PGE synthase, nitric oxide (NO), and systemic inflammation markers, indicating that it is a gut beneficial compound and exerts antioxidative and anti-inflammatory effect [34].

### 3.3. Propolis

Propolis or bee glue is a resinous substance that bees collect from various living plants for construction and adaptation of their nests, consisting of three major components: flavonoids, phenolic compounds, and caffeic acid phenethyl ester (CAPE). As a functional food, it has a range of biological activities such as anti-inflammatory, antibiotic, antioxidative, anticancer, antifungal, anesthetic, and cytostatic effects [35]. In Caco-2 cells and in rats, propolis protects intestinal barrier function by increasing expression of tight junction loci occludin and zonula occluden and activating AMP-activated protein kinase and extracellular regulated protein kinase signaling [36]. In intestinal epithelial cells, CAPE, as an active constituent of honeybee propolis, inhibits nuclear factor- (NF-) *κ*B signaling and TNF-induced and IFN-induced protein- (IP-) 10 expression, independently of p38 mitogen-activated protein kinase (MAPK) and HO-1 signaling pathways, revealing its anti-inflammatory effect [37, 38].

### 3.4. PUFAs

PUFAs, a group of unsaturated fatty acids enriched in vegetable and fish oil, functionally act as important signaling molecules regulating diverse physiological processes. They are divided into two families: omega- (*ω*-) 3 fatty acids such as alpha-linolenic acid (ALA), docosahexaenoic acid (DHA), and eicosapentaenoic acid (EPA) and *ω*-6 fatty acids such as linoleic acid and arachidonic acid (AA). *ω*-3 PUFAs, as an anti-inflammatory agent, can decrease colonic damage and inflammation and exert a beneficial protective effect in gut dysfunction. For example, in inflammatory bowel disease, *ω*-3 PUFAs significantly inhibit expression or production of NF-*κ*B, Cox-2, PGE-2, and leukotriene B4; induce expression of peroxisome proliferator-activated receptor-*γ* (PPAR-*γ*) in the colon; revert gut microbiota composition; and increase production of anti-inflammatory compounds like short-chain fatty acids, indicating that they are a potent anti-inflammatory agent and can maintain intestinal wall integrity and interaction with host immune cells [39, 40]. PUFAs can regulate gastrointestinal function by activating transient receptor potential ankyrin 1 (TRPA1), which is a cation channel in sensory neurons and gut tissues to function as a sensor of PUFAs to excite enteroendocrine cells and primary sensory neurons [41]. In the rat intestine, *ω*-3 PUFAs inhibit IL-15 expression or reduce IL-4-mediated permeability of intestinal mucosa to decrease proportion of TCR*αβ*+CD8*α*+CD8*β* cells and expression of TNF-*α*, IFN-*γ*, IL-4, and IL-10, indicating a protective role in gut immune barrier function or gut barrier integrity [42, 43]. In addition, *ω*-3 PUFAs (EPA and DHA) in fish oil have been proven to inhibit AA metabolism into inflammatory eicosanoids, displaying their anti-inflammatory profile or less inflammatory attribution, and *ω*-6 PUFA AA is, however, known as precursor of inflammatory eicosanoids like PGE-2 and leukotriene B-4 to benefit inflammatory process [44].

### 3.5. Ginsenosides

Ginsenosides or panaxosides, a class of natural steroid glycosides and triterpene saponins in the plant *Panax ginseng*, are broadly divided into two families based on carbon skeletons: four-ring dammarane (major component of ginsenosides), including protopanaxadiols (Rb1, Rb2, Rg3, Rh2, and Rh3) and protopanaxatriols (Rg1, Rg2, and Rh1), and oleanane. These ginsenosides have various biological effects such as anti-inflammation, antistress, anxiolytic, and antitumor, as well as protective effect on gastrointestinal dysfunction and gut barrier function. In rats with postoperative ileus, expression of TNF-*α*, IL-1*β*, IL-6, and IL-10 is reduced by Rb1 in ileum tissue, along with gastrointestinal transit increased, indicating a potent anti-inflammatory effect [45]. In jejunal stimulation, ginsenosides display a bidirectional regulation: jejunal contractility is increased in low contractile states caused by cholinergic activation, whereas the contractility is decreased in high contractile states caused by adrenergic activation and NO [46]. Ginsenoside compound K from Rb1,2 enhances glucose uptake of intestinal epithelial cells by upregulating cAMP response element-binding protein (CREB) and glucose transporter 1, as well as epidermal growth factor receptor, indicating its functional regulation and anti-inflammatory effect in gut [47]. In mice with colitis, their oral administration inhibits expression of TNF-*α*, IL-1*β*, and IL-17 and restores Th17/Treg imbalance, indicating their anti-inflammatory effect in inflammatory gut diseases [48].

Taken together, the phytochemicals can counteract inflammatory and oxidative stresses by their intrinsic ability to scavenge free radicals and maintain homeostasis of gut microbiota, gut barrier integrity, and immune barrier function. The improved gastrointestinal function can activate vagal neural reflex or gut-brain axis or microbiome-gut-brain axis by gut nutritional signals to change brain neurochemistry and behavior and to reverse neuroinflammatory pathogenesis in the brain [9, 25, 26]. Therefore, the gut nutritional mechanism of phytochemicals may have an important implication for developing microbe- or nutrition-based therapeutic strategies for neurodegenerative diseases [23].

## 4. Systemic Immunity Regulation by Phytochemicals and Its Connection with Brain Innate Immunity across the Blood-Brain Barrier

Immunosenescence or age-based immunity decline is a gradual deterioration of the immune system with natural age advancement, involving decline in production of new naive lymphocytes and functional competence of memory cell populations. This phenomenon entails increased risk and severity of diseases such as infections, chronic inflammation, autoimmunity, and cancer, especially obscure presentation of nonspecific signs and symptoms, leading to increase in prevalence of neurodegenerative diseases [49]. To acquire a therapeutic procedure, dietary intake of phytochemicals is increasingly used to enhance systemic immunity function and reverse intrinsic inflammatory pathologies in the diseased brain. The underlying mechanism is attributed to the immune system-brain communication, involving mononuclear phagocyte/immune factor infiltration into the brain or inflammatory factor release by microglia/astrocytes, i.e., a crosstalk between systemic inflammation and brain inflammatory microenvironment [49]. This communication process contains at least three approaches: (1) vagal afferent, i.e., sensory arm of inflammatory reflex; (2) disrupted BBB, or gateway reflex-compromised BBB [4, 8], or circumventricular organs, responsible for inflammatory infiltration into the brain parenchyma to activate microglia and astrocytes, or vice versa [5, 6, 21]; and (3) healthy BBB-lacking choroid plexus, allowing systemic inflammatory mediators or microbial products to directly interact with brain endothelium or microglia and exaggerate brain inflammation and sickness behaviors in neurodegenerative diseases [50].

### 4.1. Curcumin

Curcumin, an antioxidative and anti-inflammatory agent, can enhance systemic immunity by influencing expression or release of proinflammatory cytokines, ROS/RNS, TLRs, *β*-amyloid (A*β*), etc. Meta-analysis shows that curcumin supplementation significantly reduces concentration of circulating IL-6, a key regulator of the immune system, with more evident effect in patients with higher degree of systemic inflammation [51], indicating that curcumin can effectively inhibit inflammation propagation. In human dendritic cells (DCs), curcumin treatment limits DC maturation, reduces proinflammatory cytokine production, and prevents induction of allospecific T cell responses [52]. In the mononuclear cells from AD patients, curcuminoid compound reverses defective phagocytosis of A*β* and expression depression of TLRs such as TLRs 2–4, reflecting its ability to correct immune defects [53, 54]. Curcumin significantly decreases elevated production of IFN-*γ* and IL-17, upregulates secretion of IL-10 and PPAR-*γ*, and increases CD4(+)CD25(±) Foxp3(+) Treg cells in the CNS and lymphoid organs, showing its obvious immune enhancement effect [55]. In addition, curcumin inhibits intracellular inflammatory pathways to control lipopolysaccharide- (LPS-) induced expression of IL-6, TNF-*α*, PGE2, and Cox-2 and reverse inhibited expression of suppressor of cytokine signaling- (SOCS-) 1 and 3 and p38 MAPK in macrophages, showing its anti-inflammatory properties in chronic inflammatory diseases [56]. In patients with migraine, combination of *ω*-3 fatty acids and nanocurcumin significantly downregulates TNF-*α* mRNA expression, along with a greater decrease in the serum level than in the gene level, but no significant differences were observed in both single groups [57]. Taken together, curcumin is an effective immune enhancer to modulate systemic inflammation and brain pathologies through multiple communication mechanisms and is hopefully a particularly promising natural agent in fighting the ravages of aging and neurodegenerative diseases.

### 4.2. Resveratrol

Resveratrol, a well-known anti-inflammatory, antioxidant, immunomodulatory, and anticarcinogenic agent, can promote immune surveillance and reduce immunosenescence in rodents and humans. Evidence shows that resveratrol modulates transcription factors AP-1, NF-*κ*B, and gene Cox-2 to reduce secretion of proinflammatory cytokines (e.g., IL-6, IL-8, and TNF-*α*) and expression of adhesion proteins (e.g., intercellular adhesion molecule-1, ICAM-1) and leukocyte chemoattractants (e.g., monocyte chemotactic protein-1, MCP-1), whereas it increases production of anti-inflammatory cytokines such as IL-10 [58]. In human macrophages, *trans*- and *cis*-resveratrol can inhibit production and secretion of IL-1*β*, purinergic receptor (P2X-7R), ER stress marker (Glc-regulated protein 78), ROS, Cox-2, and PGE-2 [59]. In peripheral blood mononuclear cells (PBMCs) stimulated with LPS, resveratrol significantly inhibits production of cytokines (TNF-*α*, IL-1*α*, IL-1*β*, IFN-*γ*, IL-10, and IL-1Ra), chemokines (C-C motif ligand 2 (CCL2), CCL5) and differentiation factors such as colony-stimulating factors (CSFs), indicating its potent anti-inflammatory, antioxidant, and immunomodulatory properties [60].

### 4.3. Propolis

Propolis has a wide spectrum of pharmacological activities such as anti-inflammation and antioxidation in systemic immunity, which are principally attributed to presence of flavonoids, phenolic compounds, and CAPE [61]. Studies show that propolis increases phagocytosis and expression of IL-1*β*, IL-6, TLR2, and TLR4 in peritoneal macrophages and cytotoxicity of natural killer (NK) cells, reflecting that it potentiates cellular immunity and activates initial steps of immune response [62–64]. Brazilian red propolis downregulates TLR2 and TLR4 signaling and attenuates production of proinflammatory mediators IL-12, GM-CSF, IFN-*γ*, and IL-1*β* in LPS-induced macrophages, along with slight upregulation of TNF-*α* and IL-6 and decrease of IL-4, IL-10, and transforming growth factor- (TGF-) *β*, indicating its anti-inflammatory role [65]. CAPE, as an active component of propolis, can decrease plasma concentration of proinflammatory cytokines (e.g., TNF-*α*, IL-1*α*, IL-1*β*, IL-6, IL-4, and ICAM-1), increase anti-inflammatory cytokines (e.g., IL-10) in LPS-induced systemic inflammatory response [66], and inhibit NO production, MAPK, and NF-*κ*B signaling in mast cells and macrophages [67, 68]. In a word, comprehension of relationship between propolis and immune system has progressed in recent years, but its applicability to human health and action mechanism are not completely understood.

### 4.4. PUFAs

PUFAs, a group of immunomodulatory agents, function differently based on their families and cell contexts. In *ω*-3 PUFAs, EPA and DHA, as well as their bioactive derivatives (e.g., resolvins, protectins, and maresins), possess powerful anti-inflammatory and antioxidative effect in various inflammatory diseases, cardiovascular diseases, and cancer. AA in *ω*-6 PUFAs is generally known to be proinflammatory because it can be metabolized into PGE-2 and leukotriene B-4 by Cox and lipoxygenase enzymes, leading to production of proinflammatory eicosanoids and cytokines, whereas it is required for cell membrane fluidity and flexibility in the immune system and nervous system [69]. Moreover, PGE-2 also displays its anti-inflammatory profile by binding to one of its receptors, PGE receptor 4 (EP4), to suppress release of cytokines and chemokines in macrophages and T cells, and participates in innate and adaptive immunity and tissue repair [69]. Interestingly, in the spleen of transgenic mice overexpressing *ω*-3 fatty acid desaturase that converts *ω*-6 to *ω*-3 fatty acids, expression of IL-17, IL-6, and IL-23 is decreased, and Treg cells are expanded, along with Treg cell differentiation significantly higher, indicating an anti-inflammatory effect of *ω*-3 fatty acids [70]. Therefore, PGE-2 or AA is a dual regulator of inflammatory response due to binding to their different target receptors [69], and *ω*-3 PUFAs are well-established as potent immune nutritional agents, leading to positive clinical results and promising health promotion [71], and hopefully as an alternative therapeutic approach to impact systemic innate immunity and to control inflammatory process.

### 4.5. Ginsenosides

Ginsenosides play a critical role in regulating immune responses in inflammatory and immune-related diseases. In LPS-stimulated macrophages, ginsenoside Rg1 and its metabolites inhibit NF-*κ*B activation, expression of TNF-*α* and IL-1*β*, and phosphorylation of TGF-*β*-activated kinase 1 and IL-1 receptor-associated kinase, along with PI3K/Akt/mTOR pathways activated [48, 72], indicating their anti-inflammatory and enteric nutritional effects. In mice with cecal sepsis, Rg1 enhances the innate immunity by suppressing proinflammatory response and promoting bacterial clearance [73]. In a rat with postoperative ileus, ginsenoside Rb1 reduces serum concentration of TNF-*α*, IL-1*β*, IL-6, and IL-10, indicating its anti-inflammatory effect [45]. A ginsenoside derivative, 20S-dihydroprotopanaxadiol, upregulates functional activities of macrophages/monocytes in the innate immunity by promoting phagocytic uptake capability and generation of ROS [74].

Taken together, better understanding of immune enhancement mechanisms of phytochemicals and their relevant communication routes between the periphery and the brain, is essential to develop preventive strategies to counteract impact of systemic inflammation on brain activities in older adults, especially those with preclinical neurodegenerative diseases. The phytochemicals have a direct beneficial effect on the peripheral immune system, and so their dietary administration is an optimal choice to ameliorate systemic inflammation and then reverse pathogenesis of neurodegenerative diseases [50].

## 5. Brain Innate Immunity Regulation by Phytochemicals and Its Neuroprotective Implication

Neurodegenerative diseases are characterized by progressive neuronal loss and impaired neuronal function, along with activation of microglia and astrocytes and increased release of a range of proinflammatory cytokines such as TNF-*α*, IL-6, IL-1*β*, and IFN-*γ* and oxidative factors such as ROS, NOS, and PGE2 [5, 15, 18]. Targeting the pathogenesis of these diseases, dietary administration of phytobioactive compounds is an effective nutritional intervention choice due to their considerable antioxidant and anti-inflammatory properties [75]. It is also noted that in the striatum of PD brain, dietary administration only improves recovery and regeneration of dopamine terminals, rather than prevents their initial damage [76]. Also, the data shown in the following are largely established from *in vitro* studies, and their limited bioavailability and inability to detect them (e.g., curcumin) in the circulation or target tissues have hindered development of their therapeutic role in humans to some degree.

### 5.1. Curcumin

Curcumin, as a food additive with antioxidative and anti-inflammatory properties, has a neuroprotective effect in neurodegenerative diseases. Accumulated evidence shows that curcumin can prevent *α*-synuclein aggregation in PD, attenuate ROS-induced Cox-2 expression in ALS, ameliorate symptoms of MS and other brain injuries, and also suppress overexpression of inflammatory mediators in neuroinflammation. For example, in transgenic mice with AD, curcumin effectively counteracts p25-mediated glial activation and proinflammatory chemokine/cytokine production [10], reduces oxidative damage such as amyloid plaque burden and preformed A*β* fibrils, and reverses progression of tau/amyloid pathology, along with amelioration of cognitive impairments [77]. In prooxidant conditions of neuron culture, curcumin exerts a strong neuroprotective effect and prevents neurotoxicity of oxidative agents H_2_O_2_ and Fe^+3^ by slowing down tau aggregation and disassembling tau pathological oligomeric structures [78]. In an LPS-induced PD model, curcumin inhibits astrocyte activation and NADPH oxidase complex activation by downregulating NF-*κ*B activity, intrinsic apoptotic pathway (Bax, Bcl-2, caspase 3, and caspase 9), proinflammatory cytokines (TNF-*α*, IL-1*β*, and IL-1*α*), and inducible NOS [79]. In primary neuron culture, curcumin effectively inhibits TNF-*α*-induced neuroinflammation (IL-6 and Cox-2), protects neurons from excessive ROS production and cellular apoptosis, and promotes expression of antioxidative enzymes HO-1, catalase, and SOD-2 [80]. Recently, conjugated curcumins such as nanocurcumin or curcumin-like analogs are developed to increase their bioavailability and potential neuroprotective efficacy in PD. Although curcumin therapeutic effect for neurodegenerative diseases has been increasingly studied, its *in vivo* metabolic evidence is still not fully reported, including its pharmacokinetics, metabolism, safety, tolerance, bioavailability, and even its entry across the BBB.

### 5.2. Resveratrol

Resveratrol, as a neuroprotective agent, can suppress overexpression of inflammatory mediators in activated microglia and astrocytes. In LPS-induced cortical neurotoxicity, resveratrol significantly protects cortical neurons against neuroinflammation by inhibiting microglia activation and subsequent production of proinflammatory and cytotoxic factors such as TNF-*α*, NO, and IL-1*β* [81]. In mice with intracerebral hemorrhage, resveratrol treatment attenuates acute neurological deficits, neurodegeneration, and cerebral edema with concomitant reduction in IL-1*β* expression [82]. In LPS-induced neuroinflammation *in vivo* and *in vitro*, resveratrol suppresses microglia activation by promoting microglia polarization from proinflammatory M1 toward anti-inflammatory M2 phenotype via PGC-1*α* and reduces inflammatory damage and sickness behavior of mice [83]. Interestingly, in BBB-disrupted mice, dietary supplementation of resveratrol markedly reduces BBB-crossing lymphocytes, protein IL-17A, and matrix metalloproteinases (a tight junction degradation protein); enhances tight junction proteins; and improves BBB integrity [84]. In primary mouse astrocytes, resveratrol inhibits LPS-induced production of NO, TNF-*α*, IL-1*β*, IL-6, and MCP-1, as well as production of IL-12p40 and IL-23 (a phenotype of T cells) and C-reactive protein, indicating its anti-inflammatory and antioxidative roles in brain innate or adaptive immunity in chronic inflammatory disorders [11]. Whole-genome microarray analysis shows that in rhesus monkeys with high-fat/high-sugar (HFS) stress, dietary administration (2 years) of resveratrol differentially modulates a number of genes and pathways linked to vascular health and inflammation in cerebral cortices and ameliorates neuroinflammatory process such as oxidative stress and NF-*κ*B activation, indicating that long-term resveratrol intake elicits neuroprotective effects [85]. A robust epidemiological study indicates that moderate intake of red wine rich in resveratrol can counteract oxidative stress and metal ion deregulation produced by amyloid and metal dysmetabolism in the AD brain [86]. Collectively, resveratrol is an active scavenger of free radicals and a modulator of prosurvival or proinflammatory signaling pathways, with a greater potential for therapeutic success in counteracting neurodegenerative diseases.

### 5.3. Propolis

Propolis has been confirmed to have neuroprotective effects. In the microglia treated by hypoxia, propolis significantly inhibits expression of proinflammatory cytokines such as IL-1*β*, TNF-*α*, and IL-6; generation of ROS from mitochondria; and activation of NF-*κ*B [12]. Likewise, CAPE also inhibits expression of NOS and Cox-2 and production of NO and increases expression of HO-1 and erythropoietin (EPO) in microglia, revealing a potent antineuroinflammatory effect [87]. In kainic acid-induced excitotoxicity, propolis supplementation significantly prevents increase of NOS, NO, TNF-*α*, and caspase-3 in the rat brain, reflecting its neuroprotective role in neurodegenerative disorders [61]. In rats with PD, CAPE improves scavenging of ROS and metal chelation and inhibits mitochondrial permeability transition (MPT), a mediator of neuronal death that triggers cytochrome c release and caspase-3 activation, but has no effect on brain mitochondrial function, suggesting that CAPE is a potential compound to protect dopaminergic neuronal loss in neurodegenerative diseases [88, 89]. Also, chrysin, another component of propolis, significantly inhibits expression of NF-*κ*B, NO, inducible NOS, and Cox-2 and release of proinflammatory cytokines (TNF-*α* and IL-1*β*) [90]. Intriguingly, intragastric administration of propolis significantly inhibits acetylcholinesterase (AChE) activity in the hippocampus of scopolamine-treated mice, indicating that propolis may protect brain function through vagal reflex [91].

### 5.4. PUFAs

PUFAs, the structural component of cellular membrane in the brain, are a source of bioactive lipid mediators enzymatically derived from DHA of *ω*-3 PUFAs or AA of *ω*-6 PUFAs. DHA and AA are highly susceptible to free radical attack on microglia and astrocytes, and brain lipid metabolism relies on complex integration of diet, peripheral metabolism such as in the liver and blood, and entry of PUFAs into the brain, all of which are readily affected by genetics, sex, and aging [92, 93]. Therefore, habitual supplementation of PUFAs is an effective approach to maintain brain lipid homeostasis or counteract lipid metabolic disturbance in brain aging and neurodegenerative diseases. Evidence shows that PUFAs can be converted into essential membrane phospholipids and second messengers to modulate inflammatory response, oxidative stress, and neuronal function [94] and also regulate molecular signaling of brain innate immunity, especially in neuroinflammation and behavior disorders [92]. *ω*-3 PUFAs are important for regulating metabolic and inflammatory pathways or pleiotropic pathological activities of the brain, and DHA is especially found in high concentration in the brain (about 40% of neural phospholipids in plasma membrane). In IL-1*β*-injected rats, dietary supplementation of EPA improves *ω*-3/*ω*-6 PUFA imbalance, inhibits glial activation, reduces expression of amyloid precursor protein (APP) and TNF-*α*, and upregulates expression of BDNF and its receptor, indicating that EPA is an important candidate for anti-inflammatory therapy of neurodegenerative diseases [93]. In LPS-stimulated microglia, EPA and DHA suppress production of proinflammatory cytokines TNF-*α* and IL-6 and NF-*κ*B expression by activating Sirt1 pathways [95]. In both healthy aged adults and AD patients, cognitive function declines along with DHA decrease, and higher dietary intake of DHA and higher concentration of plasma DHA can reduce the risk of cognitive impairment or AD [96]. In healthy elderly population, DHA administration can enhance learning and memory function but cannot benefit the patients with AD progression diagnosed already [97]. In AD rat models, DHA deficiency activates caspases and exacerbates decline of glutamatergic transmission in learning and memory function [98], and this phenomenon can be reversed by DHA supplementation, along with accumulation of neuronal A*β* and tau protein reduced [99]. By contrast, AA, an *ω*-6 PUFA, can be metabolized by Cox and lipoxygenase enzymes into proinflammatory eicosanoids and PGE-2 to stimulate cytokine production and activation of microglia and astrocytes, contributing to *α*-synuclein-mediated neurotoxicity through EP-2. In a word, brain lipid metabolism and lipid signaling pathways may be viable targets for regulating microglia/astrocyte phenotype or function and developing novel therapeutic approaches for neurologic disorders.

### 5.5. Ginsenosides

Ginsenosides, the major bioactive components of ginseng, possess multiple immunoregulatory effects, involving inhibition of neuroinflammation and oxidative stress, maintenance of neurotransmitter balance, and antiapoptosis and mitochondrial stabilization in the brain. In mice with LPS-induced depression, ginsenoside Rg3 significantly reduces plasma levels of IL-6 and TNF-*α* [100]. In mice with cognitive dysfunction, Rb1 mitigates expression of ROS, TNF-*α*, and IL-6 in the hippocampus [101]. In an ALS mouse model, Re reduces microglia activation and inhibits TLR4 signaling and proinflammatory proteins such as CD14 and TNFs [102]. In LPS-induced activation of microglia *in vitro*, Rh2 inhibits inflammatory response to LPS and prevents LPS neurotoxicity, including decreased generation of NO, TNF-*α*, IL-6, IL-1*β*, Cox-2, and inducible NOS [103]. Furthermore, in a PD mouse model, oral administration of Rg1 significantly attenuates behavior defects, loss of dopamine neurons, and abnormal ultrastructure changes and regulates activation of astrocytes and microglia by decreasing release of cytokines such as TNF-*α* and IL-1*β* in substantia nigra [104]. These data indicate that ginsenosides exhibit a potent neuroprotective effect against neuroinflammation and oxidative stress and are a promising therapeutics for PD. Taken together, beneficial or therapeutic effects of ginsenosides have been widely demonstrated by preclinical and clinical studies, with most evidence obtained from AD and PD, and better understanding of their neuroprotective properties may promote their dietary application in the patients with neurodegenerative disorders and the healthy elderly in communities in order to benefit the society.

## 6. Conclusions

**Figure 2:**
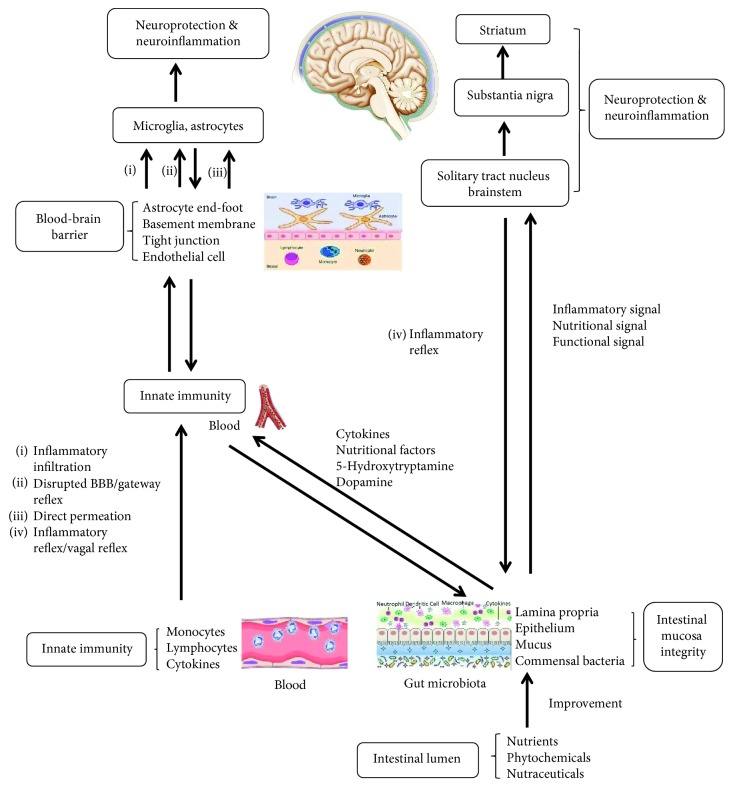
Communication between gut function, systematic immunity, and neuroinflammation. Gastrointestinal function improvement by many phytochemicals can stimulate vagal reflex to affect brain neuroinflammatory response, modulate gut functional secretion of hormones and cytokines, and facilitate systemic innate immunity, leading to neuronal functional improvement or damage reversal. There are at least four approaches connecting gut function, systematic immunity, and neuroinflammation, determining neuroinflammatory or neuroprotective outcomes through dietary intervention of the phytochemicals.

Collectively, neuroinflammation, oxidative stress, and mitochondrial dysfunction are three major situations in pathogenesis of neurodegenerative diseases (Figure 1). Nutritionally, these situations can be regulated by administration of many phytochemicals such as curcumin, resveratrol, propolis, PUFAs, and ginsenosides to mitigate inflammatory microenvironment and improve immune competency of the brain, as shown in Table 1. The phytochemicals may be an optimal therapeutic option to counteract pathogenesis of neurodegenerative diseases, mainly through four approaches (Figure 2): (1) reducing systemic inflammation by scavenging free radicals, NOS, and proinflammatory cytokines in the periphery to ease brain inflammation via the BBB; (2) reducing expression of ICAM-1 in endothelial cells to enhance integrity of disrupted BBB and inhibit inflammatory infiltration linked to gateway reflex; (3) permeating into the brain parenchyma (e.g., PUFAs) to directly act on glial cells and decrease inflammatory response of the brain; and (4) providing protective effect on gastrointestinal function and sending nutritional signals to indirectly improve brain function through gut-brain axis or vagal reflex. In addition, combination application of phytochemicals has a synergistic effect, but their efficacy, bioavailability, metabolism, and safety remain to be fully clarified. Therefore, clinical development of phytochemicals may be a novel therapeutic strategy that only alteration of lifestyle and dietary intake habit becomes an effective alternative of routine drugs to reverse systemic inflammation, oxidative stress, and neuropathologies of neurodegenerative diseases.

## Figures and Tables

**Table 1:** Anti-inflammatory or antioxidative mechanisms of several phytochemicals in gastrointestinal health, systemic immunity, and neuroimmunity.

Phytochemicals	Approaches	Action mechanisms	Major outcomes	References
Curcumin	Gastrointestinal health	BiP ↓ and IL-8 ↓, in IECs	Anti-inflammation ↑ and ER stress ↓	[27, 28, 29, 30, 31]
		Serotonin ↓, BDNF ↓, and pCREB ↓, in gut	Gut function ↑	
		Mesenteric afferent nerve response by colorectal distension or capsaicin ↓	Gut nociception ↓	
		NO ↓, lipid peroxides ↓, neutrophils infiltration ↓, and cell apoptosis ↓, in TNF-*α*-colitis	Antioxidation ↑	
		Naïve CD4(+) T cells differentiation ↑, Treg ↑, and IL-10-producing Tr1 cells ↑, in intestine	Intestinal lamina propria immunity ↑	
	Systemic immunity	Circulating IL-6 ↓, DC maturation ↓, proinflammatory cytokine ↓, and allospecific T cell response ↓	Systemic inflammation ↓	[51, 52, 53, 54, 55, 56]
		Monocyte phagocytosis of A*β* ↑ and TLRs 2–4 ↑, in AD	Systemic immunity ↑	
		IL-6 ↓, TNF-*α* ↓, IFN-*γ* ↓, IL-17 ↓, Cox-2↓, IL-10 ↑, and Treg cells ↑, in lymphoid organs or macrophages.	Anti-inflammation ↑ and innate immunity ↑	
	Neuroimmunity	Glial activation ↓, NF-*κ*B↓, TNF-*α* ↓, IL-1*β* ↓, IL-1*α* ↓, IL-6 ↓; inducible NOS ↓, Cox-2 ↓; Bax↓, Bcl-2 ↓, caspase 3↓, and caspase 9↓, in AD and PD models	Anti-inflammation ↑, antioxidation ↑, and antiapoptosis ↑	[10, 77, 78, 79, 80]
		Tau aggregation ↓ and neurotoxicity ↓, in neurons	Neuroprotective effect ↑	

Resveratrol	Gastrointestinal health	Occludin ↑ and zonula occluden (ZO-1) ↑, in IECs	Intestinal mucus integrity ↑	[32, 33, 34]
		ROS accumulation ↓, SOD ↑, and HO-1 ↑	Antioxidation ↑	
		T helper cells ↓, Treg cells ↑, and IEC proliferation ↑, in ileitis	Gut barrier function ↑ and microbiota dysbiosis ↓	
		Lactobacilli ↑, bifidobacteria ↑, and enterobacteria ↓	Colonic mucosa architecture ↑	
		PGE-2 ↓, Cox-2 ↓, PGE synthase ↓, and NO ↓, in colonic mucosa	Antioxidation ↑ and anti-inflammation ↑	
	Systemic immunity	Cytokines (TNF-*α*, IL-1*α*, IL-1*β*, IFN-*γ*, IL-6, IL-8, and IL-10) ↓, chemokines (C-C motif ligand 2 (CCL2), CCL5) ↓, ROS ↓, Cox-2 ↓, PGE-2 ↓, ICAM-1 ↓, and CSFs ↓, in monocytes and macrophage	Antioxidation ↑ and anti-inflammation ↑	[58, 59, 60]
	Neuroimmunity	Glial activation ↓, NF-*κ*B ↓, and cytotoxic factors (TNF-*α*, NO, IL-1*β*, IL-6, and C-reactive protein) ↓	Neuroprotective effect ↑, on cortical neurons	[11, 81, 84, 85, 86]
		Lymphocyte infiltration ↓, protein IL-17A ↓, matrix metalloproteinases, ↓, and tight junction proteins ↑, in BBB-disrupted mice	BBB integrity ↑	

Propolis (flavonoids, CAPE, or chrysin)	Gastrointestinal health	Occludin ↑, ZO-1 ↑ and colon fibrosis ↓, in IECs	Epithelial barrier function ↑	[35, 36, 37, 38]
		NF-*κ*B↓, proinflammatory cytokines ↓, and IP-10 ↓	Antioxidation ↑ and anti-inflammation ↑	
	Systemic immunity	Phagocytosis↑ and cytotoxicity (IL-1*β*, IL-6, TLR-2, and TLR-4) ↑, in peritoneal macrophages	Cellular immunity ↑	[62, 64, 65, 66, 67, 68]
		Circulating proinflammatory cytokines (TNF-*α*, IL-1*α*, IL-1*β*, IL-6, IL-4, and ICAM-1) ↓ and anti-inflammatory cytokines (IL-10) ↑, in LPS-induced systemic inflammation	Systemic inflammation ↓	
		NO ↓, MAPK ↓, and NF-*κ*B ↓, in mast cells and macrophages	Antioxidation ↑ and anti-inflammation ↑	
	Neuroimmunity	NF-*κ*B ↓, TNF-*α* ↓, IL-1*β* ↓, IL-6 ↓, NOS ↓, NO ↓, ROS ↓, Cox-2 ↓, and caspase-3 ↓, in microglia or PD mice	Antioxidation ↑, and anti-inflammation ↑, for neurons	[12, 61, 87, 88, 89, 90, 91]

PUFAs (*ω*-3 PUFAs)	Gastrointestinal health	NF-*κ*B ↓, Cox-2 ↓, PGE-2 ↓, and leukotriene B4 ↓	Anti-inflammation ↑, in gut	[39, 40, 41, 42, 43]
		TRPA1 activation ↑	Gastrointestinal function ↑	
		Intestinal mucosa permeability ↓, gut microbiota ↑, IL-15 ↓, TNF-*α* ↓, IFN-*γ* ↓, IL-4 ↓, and IL-10 ↓	Gut immune barrier function ↑	
	Systemic immunity	IL-17 ↓, IL-6 ↓, IL-23 ↓, and Treg cells ↑, in spleen	Anti-inflammation ↑ and immune function ↑	[69, 70, 71]
	Neuroimmunity	Glial activation ↓, *ω*-3/*ω*-6 PUFA balance ↑, amyloid precursor protein (APP) ↓, NF-*κ*B ↓, IL-6 ↓, TNF-*α* ↓, BDNF, and its receptor ↑	Neuroprotection ↑, anti-inflammation ↑, and brain innate immunity ↑	[93, 94, 95, 96, 97, 98]

Ginsenosides (Rb1, Rb2, Rg3, Rh2, Rh3, Rg1, Rg2, and Rh1)	Gastrointestinal health	TNF-*α* ↓, IL-1*β* ↓, IL-6 ↓, IL-17 ↓, IL-10 ↓, CREB ↑, glucose transporter 1 ↑, and gut contractility ↑	Anti-inflammation ↑ and gastrointestinal function ↑	[45, 46, 47, 48]
	Systemic immunity	NF-*κ*B ↓, TNF-*α* ↓, IL-1*β* ↓, and PI3K/Akt/mTOR pathways ↑	Anti-inflammation ↑ and enteric nutrition ↑	[45, 48, 72, 73, 74]
		Phagocytic uptake ↑ and ROS generation ↑	Innate immunity ↑	
	Neuroimmunity	Glial activation ↓, ROSs ↓, TNF-*α* ↓, and IL-6 ↓, in the hippocampus	Anti-inflammation ↑ and antioxidation ↑	[100, 101, 102, 103, 104]
		CD14 ↓, NO ↓, TNF-*α* ↓, IL-6 ↓, IL-1*β* ↓, Cox-2 ↓, and inducible NOS ↓, in microglia		

Notes: ↑: increased; ↓: decreased; IECs: intestine epithelial cells; abbreviations are shown in the text.

## Abbreviations

AA: Arachidonic acid
AD: Alzheimer's disease
ALA: Alpha-linolenic acid
ALS: Amyotrophic lateral sclerosis
BBB: Blood-brain barrier
BDNF: Brain-derived neurotrophic factor
CAPE: Caffeic acid phenethyl ester
CCL: C-C motif ligand
CNS: Central nervous system
Cox-2: Cycloxygenase-2
DAMPs: Damage-associated molecular patterns
DHA: Docosahexaenoic acid
EPA: Eicosapentaenoic acid
ERK: Extracellular regulated protein kinase
HD: Huntington's disease
HO-1: Heme oxygenase-1
ICAM-1: Intercellular adhesion molecule-1
IL: Interleukin
IP-10: Interferon-induced protein-10
LPS: Lipopolysaccharide
MAPK: Mitogen-activated protein kinase
MCP-1: Monocyte chemotactic protein-1
MS: Multiple sclerosis
NF-*κ*B: Nuclear factor-*κ*B
NO: Nitric oxide
NOS: Nitric oxide synthase
PAMPs: Pathogen-associated molecular patterns
PBMCs: Peripheral blood mononuclear cells
PD: Parkinson's disease
PGE-2: Prostaglandin E2
PUFAs: Polyunsaturated fatty acids
RNS: Reactive nitrogen species
ROS: Reactive oxygen species
SASP: Senescence-associated secretory phenotype
SOD: Superoxide dismutase
TGF-*β*: Transforming growth factor *β*
TLR: Toll-like receptors
TNF: Tumor necrosis factor
Treg: Regulatory T cells.

## References

[1] Fischer R., Maier O. (2015). Interrelation of oxidative stress and inflammation in neurodegenerative disease: role of TNF. *Oxidative Medicine and Cellular Longevity*.

[2] Bhullar K. S., Rupasinghe H. P. (2013). Polyphenols: multipotent therapeutic agents in neurodegenerative diseases. *Oxidative Medicine and Cellular Longevity*.

[3] Tracey K. J. (2002). The inflammatory reflex. *Nature*.

[4] Kamimura D., Ohki T., Arima Y., Murakami M. (2018). Gateway reflex: neural activation-mediated immune cell gateways in the central nervous system. *International Immunology*.

[5] Sankowski R., Mader S., Valdés-Ferrer S. I. (2015). Systemic inflammation and the brain: novel roles of genetic, molecular, and environmental cues as drivers of neurodegeneration. *Frontiers in Cellular Neuroscience*.

[6] Hernández-Romero M. C., Delgado-Cortés M. J., Sarmiento M. (2012). Peripheral inflammation increases the deleterious effect of CNS inflammation on the nigrostriatal dopaminergic system. *Neurotoxicology*.

[7] Olofsson P. S., Rosas-Ballina M., Levine Y. A., Tracey K. J. (2012). Rethinking inflammation: neural circuits in the regulation of immunity. *Immunological Reviews*.

[8] Sabharwal L., Kamimura D., Meng J. (2014). The gateway reflex, which is mediated by the inflammation amplifier, directs pathogenic immune cells into the CNS. *Journal of Biochemistry*.

[9] Daulatzai M. A. (2015). Non-celiac gluten sensitivity triggers gut dysbiosis, neuroinflammation, gut-brain axis dysfunction, and vulnerability for dementia. *CNS & Neurological Disorders Drug Targets*.

[10] Sundaram J. R., Poore C. P., Sulaimee N. H. B. (2017). Curcumin ameliorates neuroinflammation, neurodegeneration, and memory deficits in p25 transgenic mouse model that bears hallmarks of Alzheimer’s disease. *Journal of Alzheimer's Disease*.

[11] Wight R. D., Tull C. A., Deel M. W. (2012). Resveratrol effects on astrocyte function: relevance to neurodegenerative diseases. *Biochemical and Biophysical Research Communications*.

[12] Wu Z., Zhu A., Takayama F. (2013). Brazilian green propolis suppresses the hypoxia-induced neuroinflammatory responses by inhibiting NF-*κ*B activation in microglia. *Oxidative Medicine and Cellular Longevity*.

[13] Yin F., Sancheti H., Patil I., Cadenas E. (2016). Energy metabolism and inflammation in brain aging and Alzheimer’s disease. *Free Radical Biology and Medicine*.

[14] de Oliveira D. M., RMl F. L., El-Bachá R. S. (2012). Brain rust: recent discoveries on the role of oxidative stress in neurodegenerative diseases. *Nutritional Neuroscience*.

[15] Puspita L., Chung S. Y., Shim J. W. (2017). Oxidative stress and cellular pathologies in Parkinson’s disease. *Molecular Brain*.

[16] Li H., Tang Z., Chu P. (2018). Neuroprotective effect of phosphocreatine on oxidative stress and mitochondrial dysfunction induced apoptosis in vitro and in vivo: involvement of dual PI3K/Akt and Nrf2/HO-1 pathways. *Free Radical Biology & Medicine*.

[17] Zhu Y., Armstrong J. L., Tchkonia T., Kirkland J. L. (2014). Cellular senescence and the senescent secretory phenotype in age-related chronic diseases. *Current Opinion in Clinical Nutrition and Metabolic Care*.

[18] von Bernhardi R., Eugenín-von Bernhardi L., Eugenín J. (2015). Microglial cell dysregulation in brain aging and neurodegeneration. *Frontiers in Aging Neuroscience*.

[19] Currais A. (2015). Ageing and inflammation-a central role for mitochondria in brain health and disease. *Ageing Research Reviews*.

[20] Sampson T. R., Debelius J. W., Thron T. (2016). Gut microbiota regulate motor deficits and neuroinflammation in a model of Parkinson’s disease. *Cell*.

[21] Tracey K. J. (2007). Physiology and immunology of the cholinergic antiinflammatory pathway. *The Journal of Clinical Investigation*.

[22] Daulatzai M. A. (2012). Dysfunctional nucleus tractus solitarius: its crucial role in promoting neuropathogenetic cascade of Alzheimer’s dementia-a novel hypothesis. *Neurochemical Research*.

[23] Deretzi G., Kountouras J., Polyzos S. A. (2011). Gastrointestinal immune system and brain dialogue implicated in neuroinflammatory and neurodegenerative diseases. *Current Molecular Medicine*.

[24] Deretzi G., Kountouras J., Grigoriadis N. (2009). From the “little brain” gastrointestinal infection to the “big brain” neuroinflammation: a proposed fast axonal transport pathway involved in multiple sclerosis. *Medical Hypotheses*.

[25] Forsythe P., Bienenstock J., Kunze W. A. (2014). Vagal pathways for microbiome-brain-gut axis communication. *Advances in Experimental Medicine and Biology*.

[26] Daulatzai M. A. (2014). Chronic functional bowel syndrome enhances gut-brain axis dysfunction, neuroinflammation, cognitive impairment, and vulnerability to dementia. *Neurochemical Research*.

[27] Cho J. A., Park E. (2015). Curcumin utilizes the anti-inflammatory response pathway to protect the intestine against bacterial invasion. *Nutr Res Pract.*.

[28] Yu Y., Wu S., Li J. (2015). The effect of curcumin on the brain-gut axis in rat model of irritable bowel syndrome: involvement of 5-HT-dependent signaling. *Metabolic Brain Disease*.

[29] Zhi L., Dong L., Kong D. (2013). Curcumin acts via transient receptor potential vanilloid-1 receptors to inhibit gut nociception and reverses visceral hyperalgesia. *Neurogastroenterology and Motility*.

[30] Mouzaoui S., Rahim I., Djerdjouri B. (2012). Aminoguanidine and curcumin attenuated tumor necrosis factor (TNF)-*α*-induced oxidative stress, colitis and hepatotoxicity in mice. *International Immunopharmacology*.

[31] Cong Y., Wang L., Konrad A., Schoeb T., Elson C. O. (2009). Curcumin induces the tolerogenic dendritic cell that promotes differentiation of intestine-protective regulatory T cells. *European Journal of Immunology*.

[32] Wang N., Han Q., Wang G. (2016). Resveratrol protects oxidative stress-induced intestinal epithelial barrier dysfunction by upregulating heme oxygenase-1 expression. *Digestive Diseases and Sciences*.

[33] Bereswill S., Muñoz M., Fischer A. (2010). Anti-inflammatory effects of resveratrol, curcumin and simvastatin in acute small intestinal inflammation. *PLoS One*.

[34] Larrosa M., Yañéz-Gascón M. J., Selma M. V. (2009). Effect of a low dose of dietary resveratrol on colon microbiota, inflammation and tissue damage in a DSS-induced colitis rat model. *Journal of Agricultural and Food Chemistry*.

[35] González R., Ballester I., López-Posadas R. (2011). Effects of flavonoids and other polyphenols on inflammation. *Critical Reviews in Food Science and Nutrition*.

[36] Wang K., Jin X., Chen Y. (2016). Polyphenol-rich propolis extracts strengthen intestinal barrier function by activating AMPK and ERK signaling. *Nutrients*.

[37] Mapesa J. O., Waldschmitt N., Schmoeller I. (2011). Catechols in caffeic acid phenethyl ester are essential for inhibition of TNF-mediated IP-10 expression through NF-*κ*B-dependent but HO-1- and p38-independent mechanisms in mouse intestinal epithelial cells. *Molecular Nutrition & Food Research*.

[38] Khan M. N., Lane M. E., McCarron P. A., Tambuwala M. M. (2018). Caffeic acid phenethyl ester is protective in experimental ulcerative colitis via reduction in levels of pro-inflammatory mediators and enhancement of epithelial barrier function. *Inflammopharmacology*.

[39] Mbodji K., Charpentier C., Guérin C. (2013). Adjunct therapy of n-3 fatty acids to 5-ASA ameliorates inflammatory score and decreases NF-*κ*B in rats with TNBS-induced colitis. *The Journal of Nutritional Biochemistry*.

[40] Costantini L., Molinari R., Farinon B., Merendino N. (2017). Impact of omega-3 fatty acids on the gut microbiota. *International Journal of Molecular Sciences*.

[41] Motter A. L., Ahern G. P. (2012). TRPA1 is a polyunsaturated fatty acid sensor in mammals. *PLoS One*.

[42] Wang J., Zhang H., Ma H. (2008). Inhibitory effect of dietary n-3 polyunsaturated fatty acids to intestinal IL-15 expression is associated with reduction of TCRalphabeta+CD8alpha+CD8beta-intestinal intraepithelial lymphocytes. *The Journal of Nutritional Biochemistry*.

[43] Willemsen L. E., Koetsier M. A., Balvers M., Beermann C., Stahl B., van Tol E. A. (2008). Polyunsaturated fatty acids support epithelial barrier integrity and reduce IL-4 mediated permeability in vitro. *European Journal of Nutrition*.

[44] Clarke G., Fitzgerald P., Hennessy A. A. (2010). Marked elevations in pro-inflammatory polyunsaturated fatty acid metabolites in females with irritable bowel syndrome. *Journal of Lipid Research*.

[45] Tan S., Yu W., Lin Z. (2014). Anti-inflammatory effect of ginsenoside Rb1 contributes to the recovery of gastrointestinal motility in the rat model of postoperative ileus. *Biological & Pharmaceutical Bulletin*.

[46] Chen D., Xiong Y., Jiang C. (2014). Effects of ginsenosides on rat jejunal contractility. *Pharmaceutical Biology*.

[47] Wang C. W., Huang Y. C., Chan F. N. (2015). A gut microbial metabolite of ginsenosides, compound K, induces intestinal glucose absorption and Na(+) /glucose cotransporter 1 gene expression through activation of cAMP response element binding protein. *Molecular Nutrition & Food Research*.

[48] Lee S. Y., Jeong J. J., Eun S. H., Kim D. H. (2015). Anti-inflammatory effects of ginsenoside Rg1 and its metabolites ginsenoside Rh1 and 20(S)-protopanaxatriol in mice with TNBS-induced colitis. *European Journal of Pharmacology*.

[49] Perry V. H., Cunningham C., Holmes C. (2007). Systemic infections and inflammation affect chronic neurodegeneration. *Nature Reviews. Immunology*.

[50] Cunningham C., Wilcockson D. C., Campion S., Lunnon K., Perry V. H. (2005). Central and systemic endotoxin challenges exacerbate the local inflammatory response and increase neuronal death during chronic neurodegeneration. *The Journal of Neuroscience*.

[51] Derosa G., Maffioli P., Simental-Mendía L. E., Bo S., Sahebkar A. (2016). Effect of curcumin on circulating interleukin-6 concentrations: a systematic review and meta-analysis of randomized controlled trials. *Pharmacological Research*.

[52] Campbell N. K., Fitzgerald H. K., Malara A. (2018). Naturally derived heme-oxygenase 1 inducers attenuate inflammatory responses in human dendritic cells and T cells: relevance for psoriasis treatment. *Scientific Reports*.

[53] Zhang L., Fiala M., Cashman J. (2006). Curcuminoids enhance amyloid-beta uptake by macrophages of Alzheimer’s disease patients. *Journal of Alzheimer's Disease*.

[54] Fiala M., Liu P. T., Espinosa-Jeffrey A. (2007). Innate immunity and transcription of MGAT-III and Toll-like receptors in Alzheimer’s disease patients are improved by bisdemethoxycurcumin. *Proceedings of the National Academy of Sciences of the United States of America*.

[55] Kanakasabai S., Casalini E., Walline C. C., Mo C., Chearwae W., Bright J. J. (2012). Differential regulation of CD4^+^ T helper cell responses by curcumin in experimental autoimmune encephalomyelitis. *The Journal of Nutritional Biochemistry*.

[56] Guimarães M. R., Leite F. R., Spolidorio L. C., Kirkwood K. L., Rossa C. (2013). Curcumin abrogates LPS-induced pro-inflammatory cytokines in RAW 264.7 macrophages. Evidence for novel mechanisms involving SOCS-1, −3 and p38 MAPK. *Archives of Oral Biology*.

[57] Abdolahi M., Tafakhori A., Togha M. (2017). The synergistic effects of *ω*-3 fatty acids and nano-curcumin supplementation on tumor necrosis factor (TNF)-*α* gene expression and serum level in migraine patients. *Immunogenetics*.

[58] Latruffe N., Lançon A., Frazzi R. (2015). Exploring new ways of regulation by resveratrol involving miRNAs, with emphasis on inflammation. *Annals of the New York Academy of Sciences*.

[59] Huang T. T., Lai H. C., Chen Y. B. (2014). *Cis*-resveratrol produces anti-inflammatory effects by inhibiting canonical and non-canonical inflammasomes in macrophages. *Innate Immunity*.

[60] Fordham J. B., Naqvi A. R., Nares S. (2014). Leukocyte production of inflammatory mediators is inhibited by the antioxidants phloretin, silymarin, hesperetin, and resveratrol. *Mediators of Inflammation*.

[61] Swamy M., Suhaili D., Sirajudeen K. N., Mustapha Z., Govindasamy C. (2014). Propolis ameliorates tumor nerosis factor-*α*, nitric oxide levels, caspase-3 and nitric oxide synthase activities in kainic acid mediated excitotoxicity in rat brain. *African Journal of Traditional, Complementary, and Alternative Medicines*.

[62] Orsatti C. L., Missima F., Pagliarone A. C. (2010). Propolis immunomodulatory actionin vivoon Toll-like receptors 2 and 4 expression and on pro-inflammatory cytokines production in mice. *Phytotherapy Research*.

[63] Takeda K., Nagamatsu K., Okumura K. (2018). A water-soluble derivative of propolis augments the cytotoxic activity of natural killer cells. *Journal of Ethnopharmacology*.

[64] Gao W., Wu J., Wei J. (2014). Brazilian green propolis improves immune function in aged mice. *Journal of Clinical Biochemistry and Nutrition*.

[65] Bueno-Silva B., Kawamoto D., Ando-Suguimoto E. S., Alencar S. M., Rosalen P. L., Mayer M. P. (2015). Brazilian red propolis attenuates inflammatory signaling cascade in LPS-activated macrophages. *PLoS One*.

[66] Korish A. A., Arafa M. M. (2011). Propolis derivatives inhibit the systemic inflammatory response and protect hepatic and neuronal cells in acute septic shock. *The Brazilian Journal of Infectious Diseases*.

[67] Kassim M., Mansor M., Kamalden T. A. (2014). Caffeic acid phenethyl ester (CAPE): scavenger of peroxynitrite in vitro and in sepsis models. *Shock*.

[68] Cho M. S., Park W. S., Jung W. K. (2014). Caffeic acid phenethyl ester promotes anti-inflammatory effects by inhibiting MAPK and NF-*κ*B signaling in activated HMC-1 human mast cells. *Pharmaceutical Biology*.

[69] Tallima H., El Ridi R. (2017). Arachidonic acid: physiological roles and potential health benefits – a review. *Journal of Advanced Research*.

[70] Kim J. Y., Lim K., Kim K. H., Kim J. H., Choi J. S., Shim S. C. (2018). N-3 polyunsaturated fatty acids restore Th17 and Treg balance in collagen antibody-induced arthritis. *PLoS One*.

[71] Maskrey B. H., Megson I. L., Rossi A. G., Whitfield P. D. (2013). Emerging importance of omega-3 fatty acids in the innate immune response: molecular mechanisms and lipidomic strategies for their analysis. *Molecular Nutrition & Food Research*.

[72] Wang Y., Liu Y., Zhang X. Y. (2014). Ginsenoside Rg1 regulates innate immune responses in macrophages through differentially modulating the NF-*κ*B and PI3K/Akt/mTOR pathways. *International Immunopharmacology*.

[73] Zou Y., Tao T., Tian Y. (2013). Ginsenoside Rg1 improves survival in a murine model of polymicrobial sepsis by suppressing the inflammatory response and apoptosis of lymphocytes. *The Journal of Surgical Research*.

[74] Kim M. Y., Cho J. Y. (2013). 20S-dihydroprotopanaxadiol, a ginsenoside derivative, boosts innate immune responses of monocytes and macrophages. *Journal of Ginseng Research*.

[75] Joseph J., Cole G., Head E., Ingram D. (2009). Nutrition, brain aging, and neurodegeneration. *The Journal of Neuroscience*.

[76] Strömberg I., Gemma C., Vila J., Bickford P. C. (2005). Blueberry- and spirulina-enriched diets enhance striatal dopamine recovery and induce a rapid, transient microglia activation after injury of the rat nigrostriatal dopamine system. *Experimental Neurology*.

[77] Lim G. P., Chu T., Yang F., Beech W., Frautschy S. A., Cole G. M. (2001). The curry spice curcumin reduces oxidative damage and amyloid pathology in an Alzheimer transgenic mouse. *The Journal of Neuroscience*.

[78] Morales I., Cerda-Troncoso C., Andrade V., Maccioni R. B. (2017). The natural product curcumin as a potential coadjuvant in Alzheimer’s treatment. *Journal of Alzheimer's Disease*.

[79] Sharma N., Nehru B. (2017). Curcumin affords neuroprotection and inhibits *α*-synuclein aggregation in lipopolysaccharide-induced Parkinson’s disease model. *Inflammopharmacology*.

[80] Xiao L., Ding M., Fernandez A., Zhao P., Jin L., Li X. (2017). Curcumin alleviates lumbar radiculopathy by reducing neuroinflammation, oxidative stress and nociceptive factors. *European Cells & Materials*.

[81] Zhang F., Wang H., Wu Q. (2013). Resveratrol protects cortical neurons against microglia-mediated neuroinflammation. *Phytotherapy Research*.

[82] Bonsack F., Alleyne C. H., Sukumari-Ramesh S. (2017). Resveratrol attenuates neurodegeneration and improves neurological outcomes after intracerebral hemorrhage in mice. *Frontiers in Cellular Neuroscience*.

[83] Yang X., Xu S., Qian Y., Xiao Q. (2017). Resveratrol regulates microglia M1/M2 polarization via PGC-1*α* in conditions of neuroinflammatory injury. *Brain, Behavior, and Immunity*.

[84] Saha A., Sarkar C., Singh S. P. (2012). The blood-brain barrier is disrupted in a mouse model of infantile neuronal ceroid lipofuscinosis: amelioration by resveratrol. *Human Molecular Genetics*.

[85] Bernier M., Wahl D., Ali A. (2016). Resveratrol supplementation confers neuroprotection in cortical brain tissue of nonhuman primates fed a high-fat/sucrose diet. *Aging*.

[86] Granzotto A., Zatta P. (2014). Resveratrol and Alzheimer’s disease: message in a bottle on red wine and cognition. *Frontiers in Aging Neuroscience*.

[87] Tsai C. F., Kuo Y. H., Yeh W. L. (2015). Regulatory effects of caffeic acid phenethyl ester on neuroinflammation in microglial cells. *International Journal of Molecular Sciences*.

[88] Noelker C., Bacher M., Gocke P. (2005). The flavanoide caffeic acid phenethyl ester blocks 6-hydroxydopamine-induced neurotoxicity. *Neuroscience Letters*.

[89] Barros Silva R., Santos N. A., Martins N. M. (2013). Caffeic acid phenethyl ester protects against the dopaminergic neuronal loss induced by 6-hydroxydopamine in rats. *Neuroscience*.

[90] Ha S. K., Moon E., Kim S. Y. (2010). Chrysin suppresses LPS-stimulated proinflammatory responses by blocking NF-*κ*B and JNK activations in microglia cells. *Neuroscience Letters*.

[91] Chen J., Long Y., Han M., Wang T., Chen Q., Wang R. (2008). Water-soluble derivative of propolis mitigates scopolamine-induced learning and memory impairment in mice. *Pharmacology, Biochemistry, and Behavior*.

[92] Layé S., Nadjar A., Joffre C., Bazinet R. P. (2018). Anti-inflammatory effects of omega-3 fatty acids in the brain: physiological mechanisms and relevance to pharmacology. *Pharmacological Reviews*.

[93] Dong Y., Xu M., Kalueff A. V., Song C. (2017). Dietary eicosapentaenoic acid normalizes hippocampal omega-3 and 6 polyunsaturated fatty acid profile, attenuates glial activation and regulates BDNF function in a rodent model of neuroinflammation induced by central interleukin-1*β* administration. *European Journal of Nutrition*.

[94] Lukiw W. J., Bazan N. G. (2008). Docosahexaenoic acid and the aging brain. *The Journal of Nutrition*.

[95] Inoue T., Tanaka M., Masuda S. (2017). Omega-3 polyunsaturated fatty acids suppress the inflammatory responses of lipopolysaccharide-stimulated mouse microglia by activating SIRT1 pathways. *Biochimica et Biophysica Acta (BBA) - Molecular and Cell Biology of Lipids*.

[96] Yurko-Mauro K., Alexander D. D., Van Elswyk M. E. (2015). Docosahexaenoic acid and adult memory: a systematic review and meta-analysis. *PLoS One*.

[97] Chiu C. C., Su K. P., Cheng T. C. (2008). The effects of omega-3 fatty acids monotherapy in Alzheimer’s disease and mild cognitive impairment: a preliminary randomized double-blind placebo-controlled study. *Prog Neuro- psychopharmacol Biol Psychiatry.*.

[98] Calon F., Lim G. P., Morihara T. (2005). Dietary n-3 polyunsaturated fatty acid depletion activates caspases and decreases NMDA receptors in the brain of a transgenic mouse model of Alzheimer’s disease. *The European Journal of Neuroscience*.

[99] Green K. N., Martinez-Coria H., Khashwji H. (2007). Dietary docosahexaenoic acid and docosapentaenoic acid ameliorate amyloid-*β* and tau pathology via a mechanism involving presenilin 1 levels. *The Journal of Neuroscience*.

[100] Kang A., Xie T., Zhu D., Shan J., Di L., Zheng X. (2017). Suppressive effect of ginsenoside Rg3 against lipopolysaccharide-induced depression-like behavior and neuroinflammation in mice. *Journal of Agricultural and Food Chemistry*.

[101] Miao H. H., Zhang Y., Ding G. N., Hong F. X., Dong P., Tian M. (2017). Ginsenoside Rb1 attenuates isoflurane/surgery-induced cognitive dysfunction via inhibiting neuroinflammation and oxidative stress. *Biomedical and Environmental Sciences*.

[102] Cai M., Yang E. J. (2016). Ginsenoside Re attenuates neuroinflammation in a symptomatic ALS animal model. *The American Journal of Chinese Medicine*.

[103] Vinoth Kumar R., Oh T. W., Park Y. K. (2016). Anti-inflammatory effects of ginsenoside-Rh2 inhibits LPS-induced activation of microglia and overproduction of inflammatory mediators via modulation of TGF-*β*1/Smad pathway. *Neurochemical Research*.

[104] Heng Y., Zhang Q. S., Mu Z., Hu J. F., Yuan Y. H., Chen N. H. (2016). Ginsenoside Rg1 attenuates motor impairment and neuroinflammation in the MPTP-probenecid-induced Parkinsonism mouse model by targeting *α*-synuclein abnormalities in the substantia nigra. *Toxicology Letters*.

